# Supplementary material for: Modelling the Effect of MUC1 on Influenza Virus Infection Kinetics and Macrophage Dynamics
Source: Viruses. 2021 May 7;13(5):850. doi: 10.3390/v13050850 (PMC8150684; doi:10.3390/v13050850)
Supplement: Supplementary file 1 [file viruses-13-00850-s001.zip › Supplementary_Figures.pdf]

Supplementary Figures

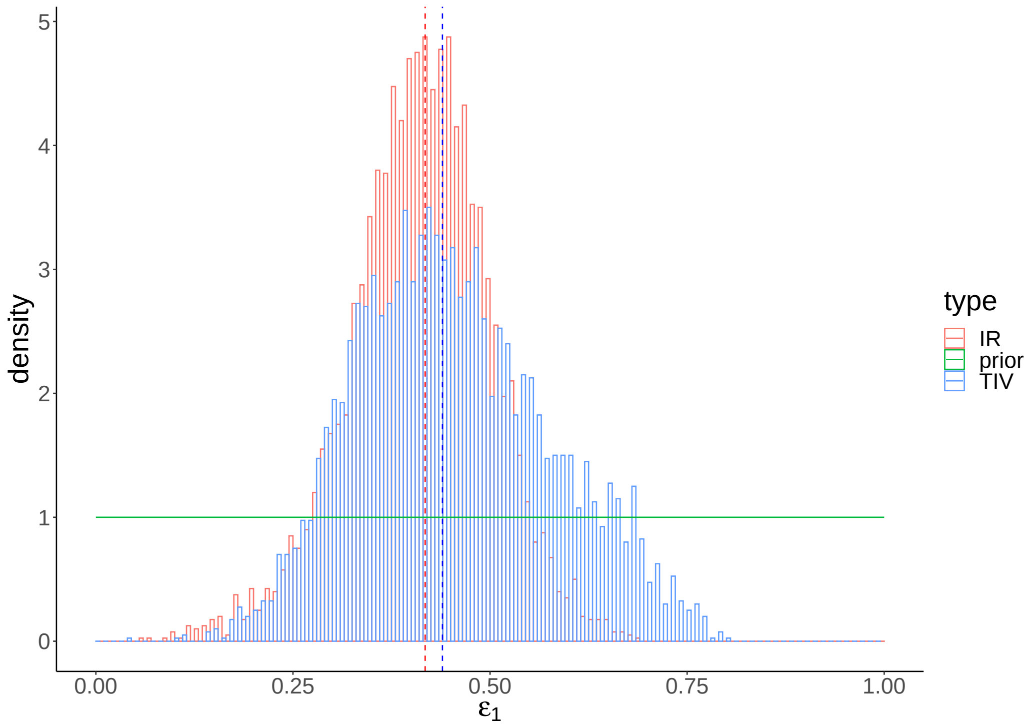

Figure S1: The prior (green) and posterior distributions for ε_1 in TIV (blue) and IR (red) models. Dashed lines indicate the posterior-median estimates. A detailed prior distribution see Table 1 in Supplementary Materials.

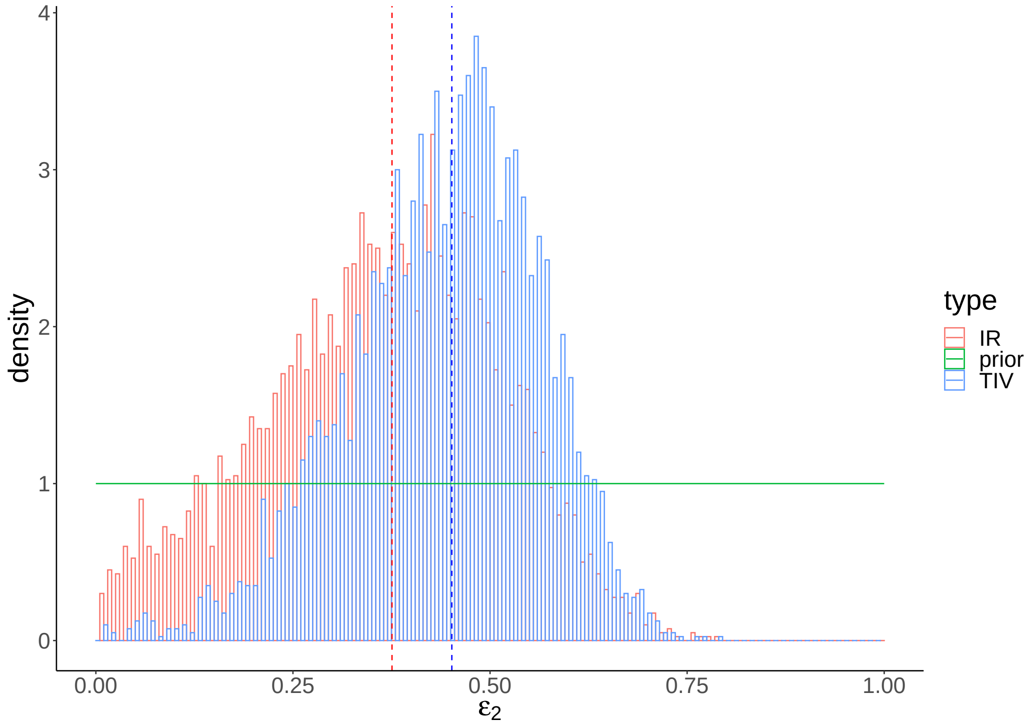

Figure S2: The prior (green) and posterior distributions for ε_2 in TIV (blue) and IR (red) models. Dashed lines indicate the posterior-median estimates. A detailed prior distribution see Table 1 in Supplementary Materials.

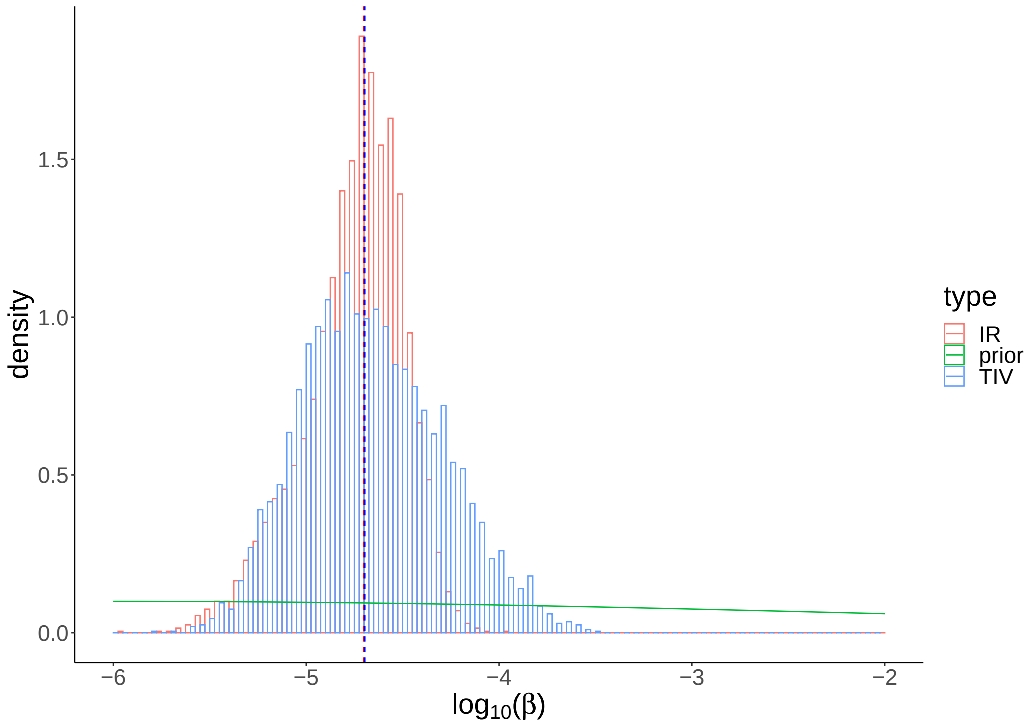

Figure S3: The prior (green) and posterior distributions for $\log_{10}(\beta)$ in TIV (blue) and IR (red) models. Dashed lines indicate the posterior-median estimates. A detailed prior distribution see Table 1 in Supplementary Materials.

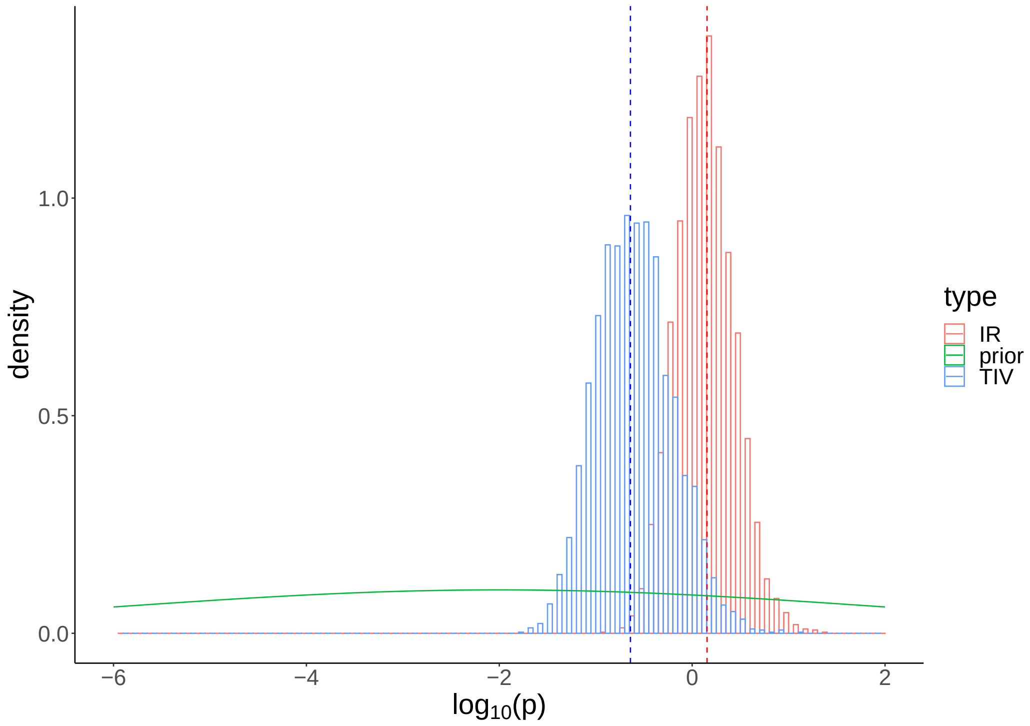

Figure S4: The prior (green) and posterior distributions for $\log_{10}(p)$ in TIV (blue) and IR (red) models. Dashed lines indicate the posterior-median esti-mates. A detailed prior distribution see Table 1 in Supplementary Materials.

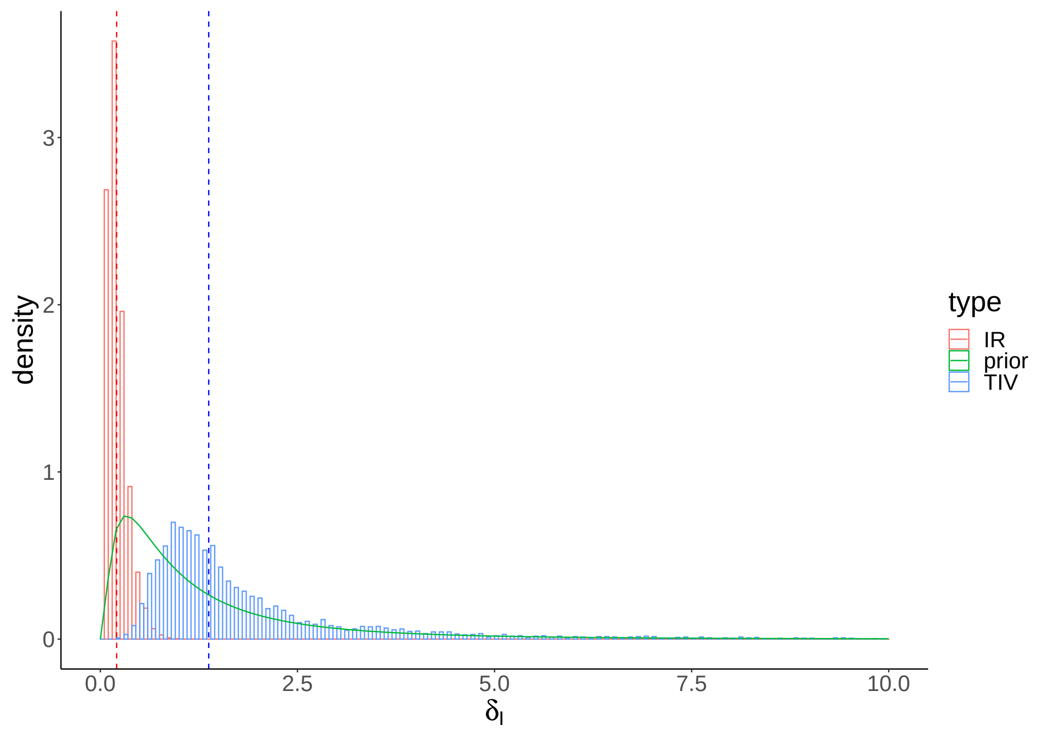

Figure S5: The prior (green) and posterior distributions for δ_I in TIV (blue) and IR (red) models. Dashed lines indicate the posterior-median estimates. A detailed prior distribution see Table 1 in Supplementary Materials.

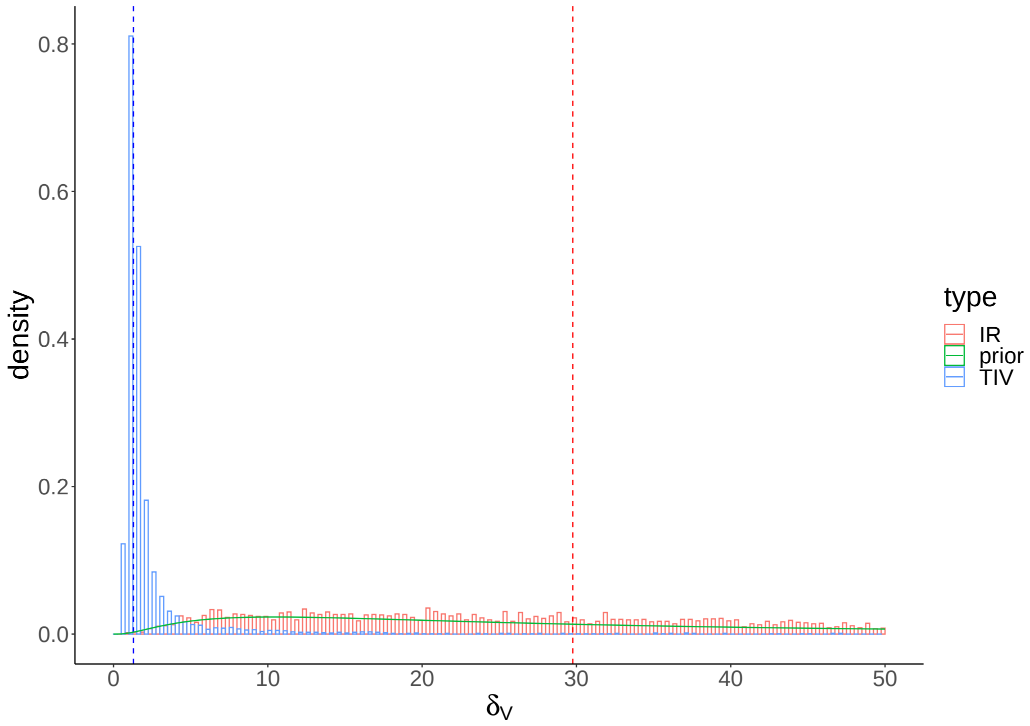

Figure S6: The prior (green) and posterior distributions for δ_V in TIV (blue) and IR (red) models. Dashed lines indicate the posterior-median estimates. A detailed prior distribution see Table 1 in Supplementary Materials.

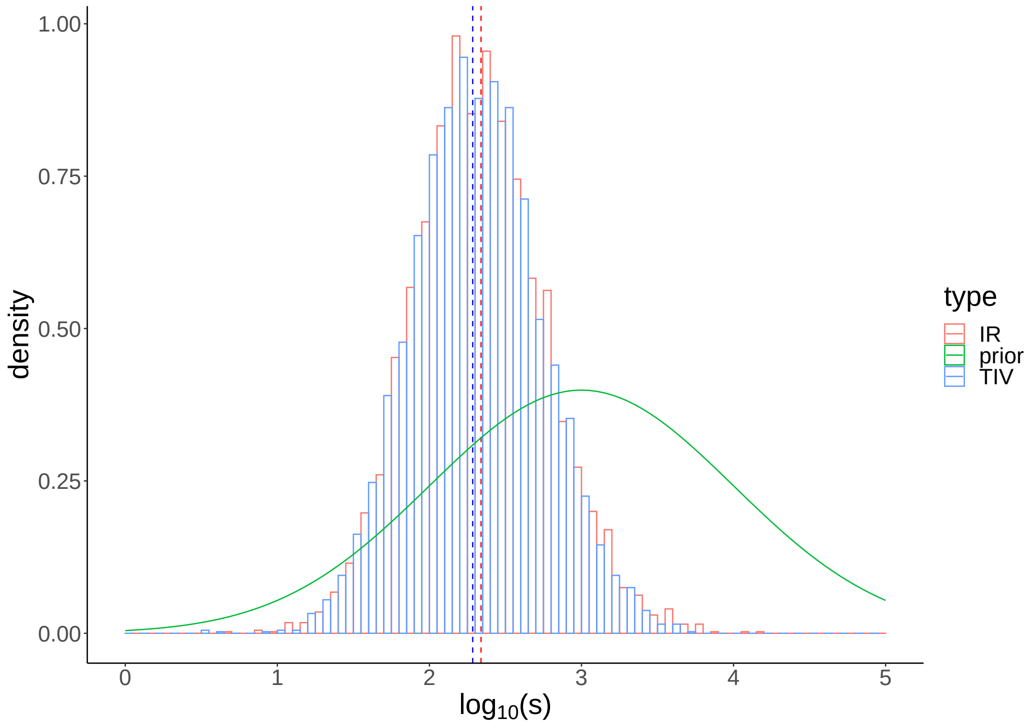

Figure S7: The prior (green) and posterior distributions for $\log(s)$ in TIV (blue) and IR (red) models. Dashed lines indicate the posterior-median esti-mates. A detailed prior distribution see Table 1 in Supplementary Materials.

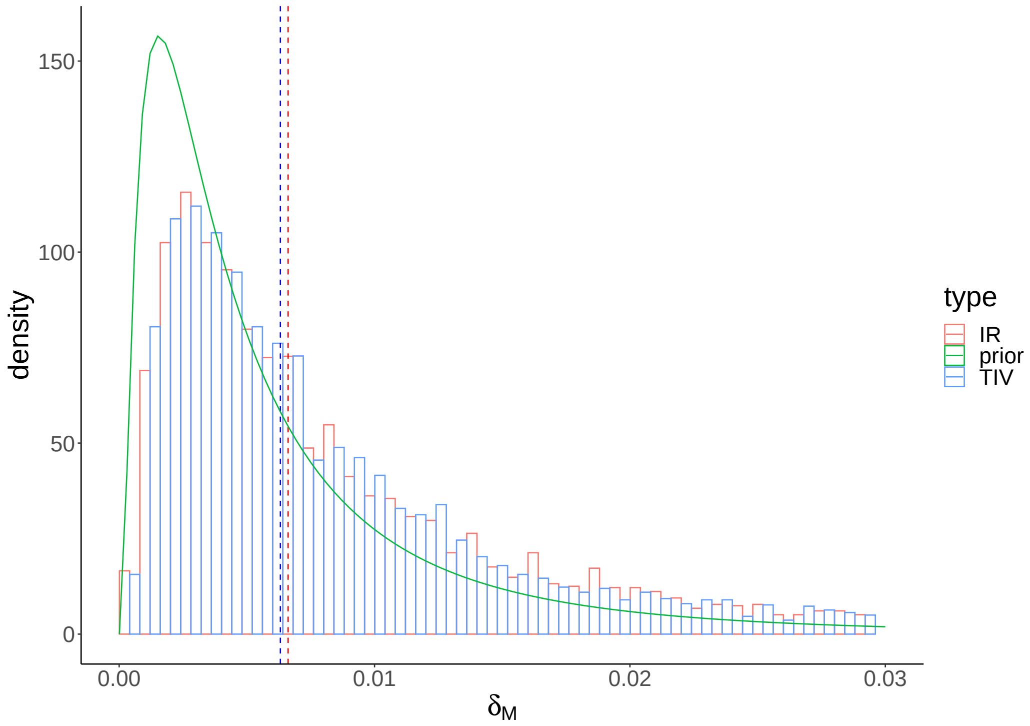

Figure S8: The prior (green) and posterior distributions for δ_M in TIV (blue) and IR (red) models. Dashed lines indicate the posterior-median estimates. A detailed prior distribution see Table 1 in Supplementary Materials.

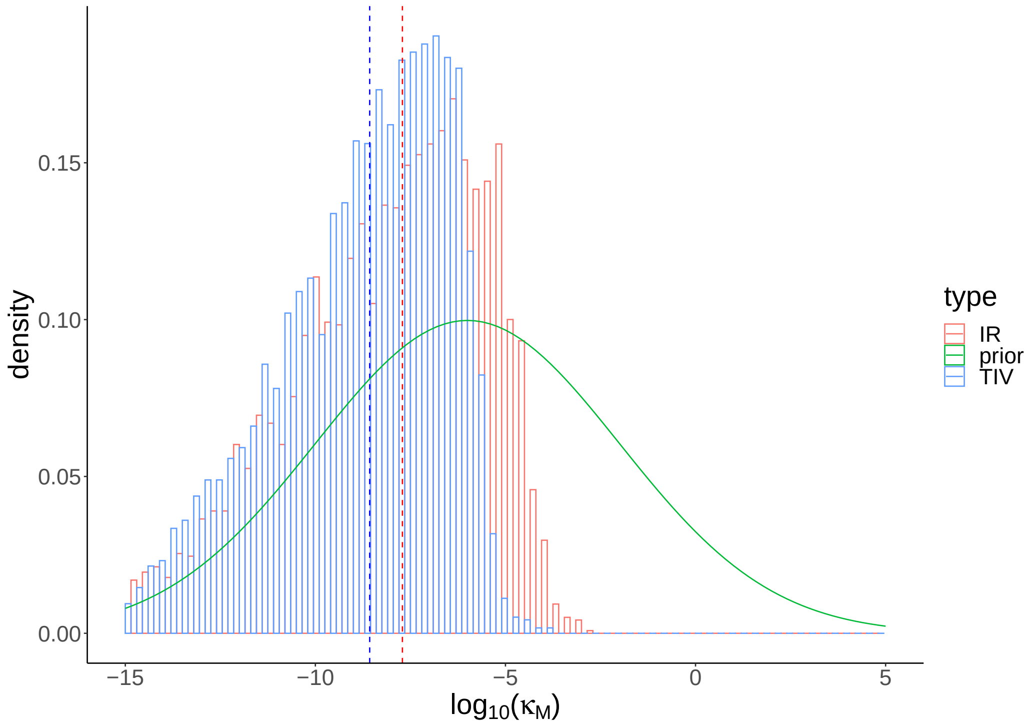

Figure S9: The prior (green) and posterior distributions for $\log_{10}(\kappa_M)$ in TIV (blue) and IR (red) models. Dashed lines indicate the posterior-median estimates. A detailed prior distribution see Table 1 in Supplementary Materials.

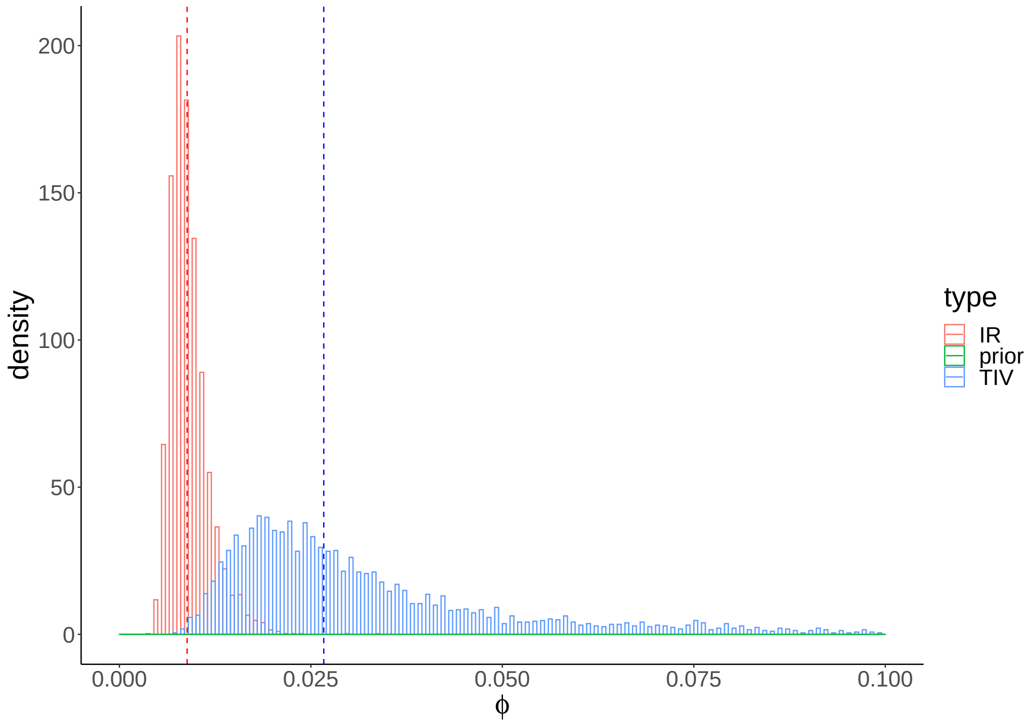

Figure S10: The prior (green) and posterior distributions for ϕ in TIV (blue) and IR (red) models. Dashed lines indicate the posterior-median estimates. A detailed prior distribution see Table 1 in Supplementary Materials.

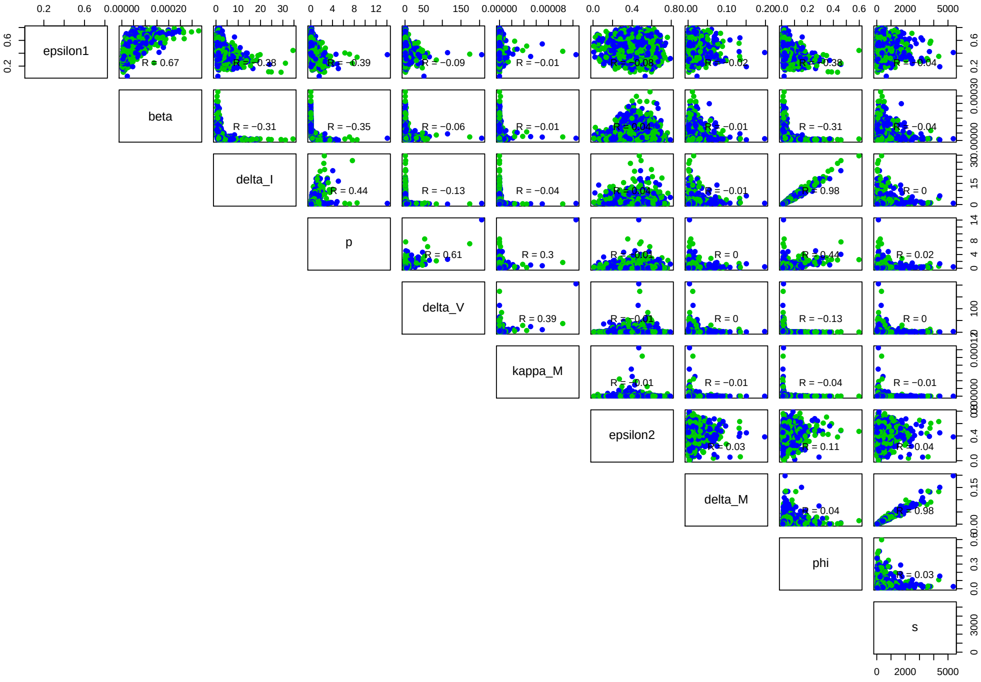

Figure S11: The correlation map for the estimated parameters in the TIV model.

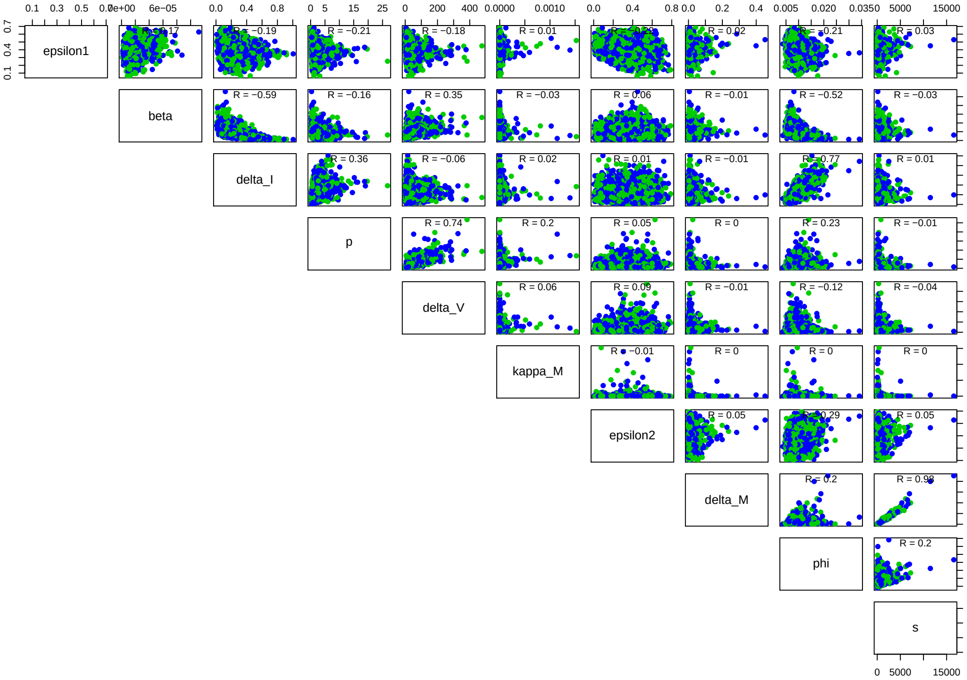

Figure S12: The correlation map for the estimated parameters in the IR model.

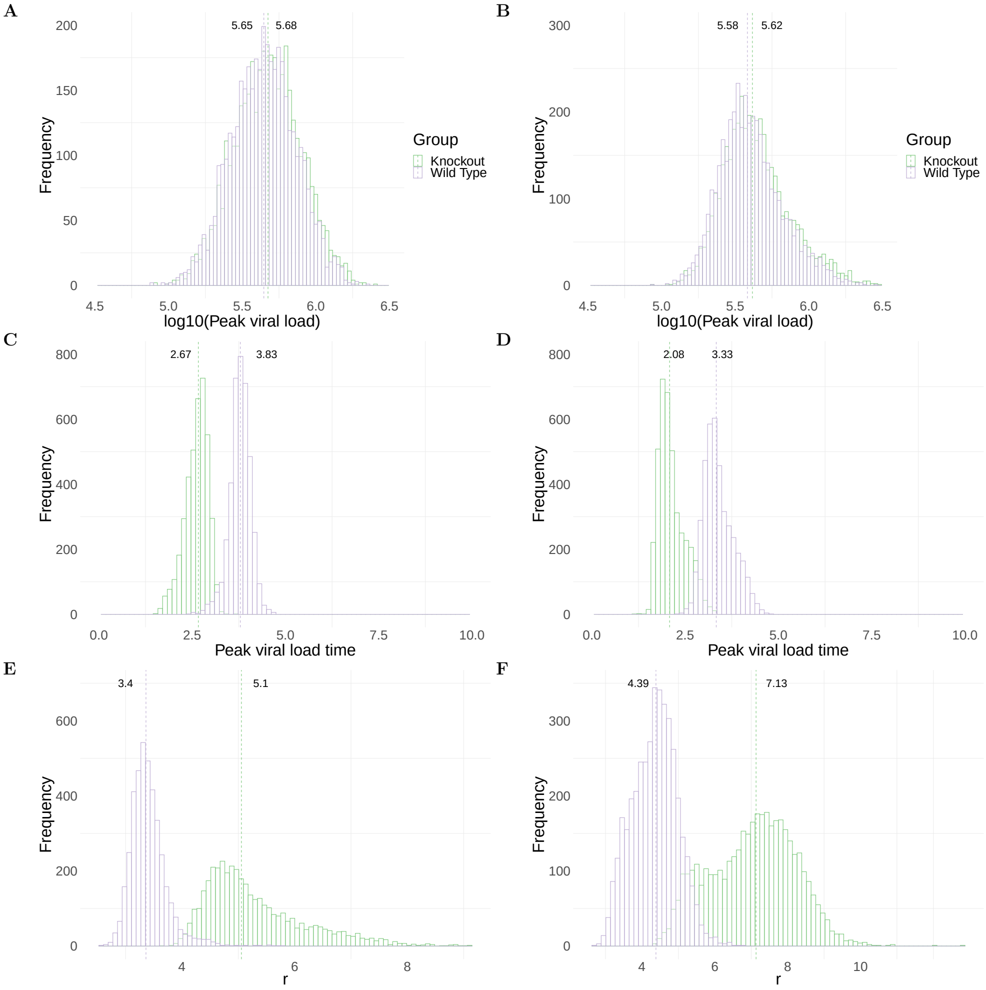

Figure S13: **Comparison of model predictions for key biological quantities**. The distribution of the quantities is calculated using 4000 joint posterior distributions through model calibration. Panels A and B show the distribution for $\log_{10}$(peak viral load) in wildtype (purple) and MUC1-knockout (green) group in TIV (left panel) and IR models (right panel), respectively. Panels C and D show the distribution for peak viral load time in different mice groups in the two models. Panels E and F show the the initial growth rate of viral replication in the two models. The initial viral regrowth rate is given by $r = (-(\delta_I + \delta_V + \kappa_M M_0) + \sqrt{(\delta_I + \delta_V + \kappa_M M_0)^2 - 4(\delta_I(\delta_V + \kappa_M M_0) - \beta p T_0)})/2$, where M_0 and T_0 are initial number of epithelial cells and macrophages, respectively.

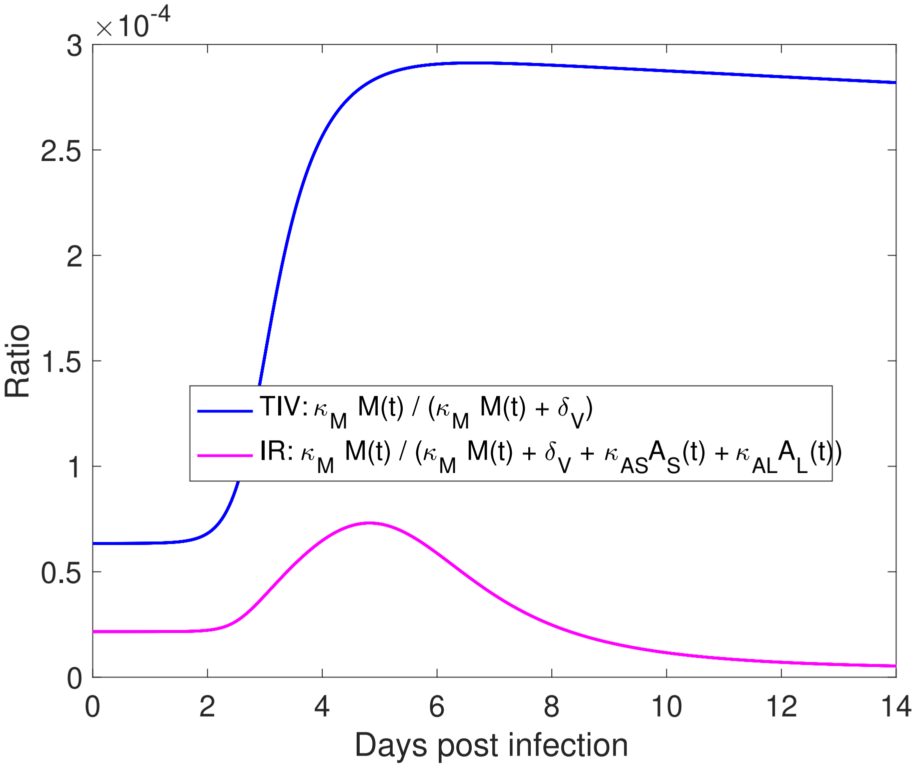

Figure S14: **Relative contribution of macrophage-mediated viral clearance in the TIV and IR models**. We used posterior-median estimates of model parameters to compute the ratio shown in the legend in the TIV (blue line) and IR (purple line) models, respectively. The value of fixed model parameters used for simulation is given in Table 2 in Supplementary Materials.
