# Supplementary material for: Modelling the Effect of MUC1 on Influenza Virus Infection Kinetics and Macrophage Dynamics
Source: Viruses. 2021 May 7;13(5):850. doi: 10.3390/v13050850 (PMC8150684; doi:10.3390/v13050850)
Supplement: Supplementary file 1 [file viruses-13-00850-s001.zip › Supplementary_Materials.pdf]

Supplementary Material 1

Model parameters and Priors

Table S1 gives parameter values for fixed parameters. Table S2 gives the prior distribu-tion for estimated parameters. Note that we estimate β, p, κ_M, s and ϕ in logarithmic space. For example, the estimated ranges of $\log_{10}(\beta)$ were (-8, -4) from literature, which indicate the estimated ranges of β were $(10^{-8}, 10^{-4})$. We set the priors which allow us to explore a wide range of biological plausible parameter values.

Par.	Description	Values [Refs]	Unit
g	Epithelial cell regrowth rate	0.8 [1, 2]	/day
γ_E	Maximal stimulation rate of naive CD8 T cells	10 [2]	/day
E_{50}	Half-maximal stimulating viral titer for CD8 T cells	1e+4	$[u_V]$
n_E	Number of effector T cell division cycle	5 [2]	division
τ_E	Total proliferation time of CD8 T cells	8 [2]	day
ϕ_E	Activation rate of matured CD8 T cells	1.4e+3 [2]	/day
δ_E	Decay rate of CD8 T cells	0.57 [2]	/day
κ_E	Lysing rate of infected cells by CD8 T cells	5e-5 [3]	/day/cell
γ_B	Maximal stimulation rate of naive B cells	6e-2 [2]	/day
B_{50}	Half-maximal stimulating viral titer for B cells	6e-2	$[u_V]$
n_B	Number of B cell division cycle	5 [2]	division
τ_B	Total proliferation time of B cells	8 [2]	day
ϕ_P	Activation rate of matured plasma cells	8 [2]	/day
δ_P	Decay rate of plasma cells	0.5 [2]	/day
μ_S	Production rate of short-lived antibody	12 [1, 2, 3]	$[u_A]$/cell/day
δ_{AS}	Decay rate of short-lived antibody	2 [1]	/day
μ_L	Production rate of long-lived antibody	4 [3]	$[u_A]$/cell/day
δ_{AL}	Decay rate of long-lived antibody	0.015	/day
κ_{AS}	Neutralisation rate of virus by short-lived antibody	0.8 [3]	$[u_A]$/day
κ_{AL}	Neutralisation rate of virus by long-lived antibody	0.8 [1, 3]	$[u_A]$/day
T_{max}	The maximal number of epithelial cells in the upper respiratory tract	1e+7 [4]	cell

Table S1: **Parameter values for fixed parameters.** $[\cdot]$ denotes the unit of variables, e.g., the unit of antibody is denoted as $[u_A]$, and the unit of virus is denoted as $[u_V]$.

Par.	Description	Estimated values from literature [Refs]	Unit	Prior
ε_1	The effect of MUC1 on viral infectivity	-	-	Uniform(0,1)
$\log_{10}(\beta)$	Rate of viral infection	(-8,-4) [4, 5]	$/([u_V]$ day)	Normal(-6,-4)
δ_I	Death rate of infected cells	(0.67, 4.8)[4, 5, 6]	/day	Lognormal(log(0.89),1)
$\log_{10}(p)$	Viral production rate	(-6,2)[4, 5, 6]	$[u_V]$/(cell day)	Normal(-2,4)
δ_V	Natural death rate of virus	(4.2, 59)[4, 5, 6]	/day	Lognormal(log(28.4),1)
$\log_{10}(\kappa_M)$	Phagocytosis rate of virus by macrophages	(-6,-3)[7]	/(cell day)	Normal(-6,4)
ε_2	The effect of MUC1 on macrophage recruitment	-	-	Uniform(0,1)
δ_M	Decay rate of macrophages	(1/180,1/150)[7]	/day	Lognormal(log(4.2e-3),1)
$\log_{10}(s)$	Supplementary rate of macrophages	(2.52, 2.63)[7]	cell/(ml day)	Normal(3,1)
$\log_{10}(\phi)$	Recruitment rate of macrophages by infected cells	-	(ml cell)/cell	Normal(0,3)

Table S2: **Priors for estimated model parameters.** $[\cdot]$ denotes the unit of variables, e.g., the unit of virus is denoted as $[u_V]$.

References

[1] P. Cao, A. W. Yan, J. M. Heffernan, S. Petrie, R. G. Moss, L. A. Carolan, T. A. Guarnaccia, A. Kelso, I. G. Barr, J. McVernon, et al., Innate immunity and the inter-exposure interval determine the dynamics of secondary influenza virus infection and explain observed viral hierarchies, PLoS Comput Biol 11 (8) (2015) e1004334.

[2] A. W. Yan, P. Cao, J. M. Heffernan, J. McVernon, K. M. Quinn, N. L. La Gruta, K. L. Laurie, J. M. McCaw, Modelling cross-reactivity and memory in the cellular adaptive immune response to influenza infection in the host, Journal of Theoretical Biology 413 (2017) 34–49.

[3] P. Cao, Z. Wang, A. W. Yan, J. McVernon, J. Xu, J. M. Heffernan, K. Kedzierska, J. M. McCaw, On the role of cd8+ t cells in determining recovery time from influenza virus infection, Frontiers in immunology 7 (2016) 611.

[4] A. M. Smith, F. R. Adler, J. L. McAuley, R. N. Gutenkunst, R. M. Ribeiro, J. A. McCullers, A. S. Perelson, Effect of 1918 PB1-F2 expression on influenza A virus infection kinetics, PLoS Comput Biol 7 (2) (2011) e1001081.

[5] P. Baccam, C. Beauchemin, C. A. Macken, F. G. Hayden, A. S. Perelson, Kinetics of influenza A virus infection in humans, Journal of virology 80 (15) (2006) 7590–7599.

[6] H. Miao, J. A. Hollenbaugh, M. S. Zand, J. Holden-Wiltse, T. R. Mosmann, A. S. Perelson, H. Wu, D. J. Topham, Quantifying the early immune response and adaptive immune response kinetics in mice infected with influenza a virus, Journal of virology 84 (13) (2010) 6687–6698.

[7] J. E. Wigginton, D. Kirschner, A model to predict cell-mediated immune regulatory mechanisms during human infection with mycobacterium tuberculosis, The Journal of Immunology 166 (3) (2001) 1951–1967.
